# Molecular Identification of Adult and Juvenile Linyphiid and Theridiid Spiders in Alpine Glacier Foreland Communities

**DOI:** 10.1371/journal.pone.0101755

**Published:** 2014-07-22

**Authors:** Lorna Raso, Daniela Sint, Alexander Rief, Rüdiger Kaufmann, Michael Traugott

**Affiliations:** Institute of Ecology, University of Innsbruck, Innsbruck, Austria; Naval Research Laboratory, United States of America

## Abstract

In glacier forelands spiders constitute a large proportion of the invertebrate community. Therefore, it is important to be able to determine the species that can be found in these areas. Linyphiid and theridiid spider identification is currently not possible in juvenile specimens using traditional morphological based methods, however, a large proportion of the population in these areas are usually juveniles. Molecular methods permit identification of species at different life stages, making juvenile identification possible. In this study we tested a molecular tool to identify the 10 most common species of Linyphiidae and Theridiidae found in three glacier foreland communities of the Austrian Alps. Two multiplex PCR systems were developed and over 90% of the 753 field-collected spiders were identified successfully. The species targeted were found to be common in all three valleys during the summer of 2010. A comparison between the molecular and morphological data showed that although there was a slight difference in the results, the overall outcome was the same independently of the identification method used. We believe the quick and reliable identification of the spiders via the multiplex PCR assays developed here will aid the study of these families in Alpine habitats.

## Introduction

Linyphiidae is the largest spider family in northern Europe with over 400 described species [1]. These spiders are widely distributed but are more diverse in colder regions [2], where they can be found wandering on snow at below-zero temperatures [3]. Therefore, it is not surprising that it is a significant group in glacier foreland communities. They are present in all areas, even close to the glacier, and may play an important ecological role, bringing nutrients into the system [4]. Their diversity means that considerable effort is required to determine the community composition. Another species-rich family of spiders regularly occurring in northern and alpine habitats are the Theridiidae which includes many species which build three-dimensional webs similar to the ones created by linyphiid spiders and which they resemble also in other traits such as body size and structure [5]–[7].

Spiders from the same species can vary greatly in size and morphology depending on the sex, so most identification keys rely on the examination of adults for identification. As sexual dimorphism can be very important, many keys have separate criteria for males and females making identification even more complex [8]. In some cases it is possible to identify juvenile linyphiid and theridiid spiders to genus but not to species. This poses a problem, as it is not always possible to identify the juveniles, which can often constitute a considerable proportion (over 70%) of the spiders caught in the field (e.g. [9]). The nature of linyphiid identification may be an inconvenience if these individuals are to be used for further analysis (e.g. molecular gut content), as the necessary manipulation to expose the sexual organs (females) can damage the abdomen and in some cases pierce the gut of smaller individuals. This can be a source of cross-contamination of samples and hinder further analysis.

DNA-based techniques can overcome these difficulties as they allow the identification of the species independent of the developmental stage of the organism [10]. An alternative means to sequencing-based DNA barcoding [11], [12] for the molecular identification of specific taxa is provided by diagnostic multiplex PCR, where a specific PCR product fragment length is indicative for a specific taxon [13], [14]. Developing species-specific primers to identify species has proven useful in many situations. It is possible to identify species in different life stages such as larvae [15] as well as eggs and pupae [16]; when the samples are damaged or only parts of the organism are available for identification e.g. shark fins [17] or when morphological identification can be difficult e.g. spiders [13]. Barrett and Hebert [18] found the mitochondrial cytochrome *c* oxidase subunit 1 gene (COI) to be a useful DNA barcoding region for spider identification.

The 5′ region of the COI gene (approx. 600 base pairs) was employed in the current study to design species-specific primers for two multiplex PCR assays, designated to identify 10 species of linyphiid and theridiid spiders that commonly occur in pioneer stages of Alpine glacier forelands. This molecular method was then used to examine the community composition of linyphiid and theridiid spiders dwelling in early and late pioneer stages of three neighbouring glacier valleys in the Austrian Alps. The results from the molecular analysis and traditional morphological identification were compared to find if the final outcome was the same using either method.

## Materials and Methods

### Field work

Three valleys in the Ötztal (Austria) were sampled: Rotmoostal (46.826 N, 11.046 E), Gaisbergtal (46.836 N, 11.057 E) and Langtal (46.803 N, 11.006 E). No specific permissions were required for the arthropod sampling within the three glacier forelands and our sampling did not involve endangered or protected species. The valleys are side by side and have a similar orientation and altitude (northwest facing and reaching a lower elevation from 2200 m above mean sea level (a.s.l.) to 2500 m a.s.l. at the glacier tongue). In each valley two areas were sampled, one closest to the glacier (early pioneer stage - area A, 0–8 years ice-free) and one further away (late pioneer stage - area B, 13–20 years ice-free). The linyphiid and theridiid sampling was concentrated over the period of nearly two weeks in July (12.07.2010–23.07.2010) in which the three valleys were intensively studied.

In each valley and area, approximately 50 pitfall traps were placed in a grid from side to side of the valley/area, with a 10 m separation between them. Spiders were collected from the pitfall traps and by actively searching the areas. Active searches were considered necessary as linyphiids are web weavers and while males will probably roam, females are more likely to remain in the webs. These searches were made beside randomly chosen traps in all areas. A total of 20 traps in Gaisbergtal and 30 traps in Langtal and Rotmoostal were searched and the spiders found accounted for over half of the total spider catch. Searches were made by setting a 1 m^2^ quadrat at the lower right hand corner of the traps (North West facing) and carefully searching the area within the quadrat for spiders. If after having overturned all the stones in the quadrat and looked in all holes and crevices (for a minimum of 20 min) no spiders were found, then the quadrat was considered finished and another was searched. It was therefore possible to associate certain species to determined areas. All individuals collected were individually frozen at −28°C in 1.5 ml reaction tubes.

### Identification and molecular analysis

We identified 168 of the adult linyphiid and theridiid spiders morphologically in 96% ethanol [5], [19]–[21]. Every spider was treated with care so as to not puncture the abdomen and not contaminate other individuals and the ethanol was renewed after each identification. All petri dishes and forceps were submerged in 96% ethanol and flamed before each new sample. Once the examination was complete the individuals were placed back in the reaction tubes and stored at −28°C.

Nine species of linyphiid and one species of theridiid spiders were found within the total catch of adult specimens. A minimum of five individuals from each identified species were DNA-extracted and sequenced, while all remaining spiders were DNA-extracted and screened using the two multiplex PCRs set up for this purpose (Table 1). DNA was extracted using a CTAB (hexadecyltrimethylammonium bromide) based extraction method. Over half of the individuals extracted had been previously examined and so contained traces of ethanol. To ensure no inhibition occurred during DNA extraction from any remaining ethanol, the tubes were left open in a laminar flow extraction chamber until all ethanol had evaporated. Once dry, the CTAB DNA-extraction protocol described by Juen & Traugott [22] was followed. Negative controls were included to check for carry-over contamination.

**Table 1:** The two primer mixes for the multiplex PCR systems.

Species	Primer	Sequence (5′ - 3′)	Product size (bp)	M1 conc. (µM)
*Mecynargus paetulus*	Mec-pae-S286	**GGGTTTTGGTAATTGATTGGTG**	127	0.4
	Mec-pae-A286	**ATCTATTCTAGAGATAAACAAAAGAAATAAC**		0.4
*Diplocephalus helleri*	Dip-hel-S278	**CCTCCTTCTTTGTTCTTACTATTTG**	151	0.2
	Dip-hel-A280	**AAGSCCCAGCCAAGTGC**		0.2
*Erigone atra*	Eri-atra-S280	**GGGCTTGGGCTGCTATAGTG**	197	0.2
	Eri-atra-A282	**CCCTAATATTAAAGGAACTAATCAGTTG**		0.2
*Janetschekia monodon*	Jan-mon-S282	**GATATTAGGAGCTCCTGATATAGCC**	240	0.2
	Jan-mon-A284	**ATAAAATTAATGGCTCCCATAATC**		0.2
*Entelecara media*	Ent-med-S279	**GAGYTAGGTCAAGTTGGAAGCC**	262	0.2
	Ent-med-A281	**TTCATCCTGCCCCAACG**		0.2
*Walckenaera vigilax*	Wal-vig-S289	**TGAGCTGCTATAGTGGGAACG**	366	0.2
	Wal-vig-A291	**AGCAAAATCTACTGAACTTCCAGAG**		0.2
*Erigone tirolensis*	Eri-tir-S281	**GGAGCTTGGGCTGCTATAGTA**	186	0.2
	Eri-tir-A283	**AGGRACTAATCAGTTACCAAAYCCT**		0.2
*Agyneta nigripes*	Agy-nig-S287	**TCAGATATAGCGTTTCCTCGTATG**	264	0.2
	Agy-nig-A288	**AGTTATACCATAGCCACGTATATTTAG**		0.2
*Robertus arundineti*	Rob-aru-S288	**TACAGCTATAAGWGTYCTAATTCGAGTA**	282	0.2
	Rob-aru-A290	**GCACCTACTCCTATTTCAACTATAGA**		0.2
*Mughiphantes variabilis*	Mug-var-S284	**TCGAATTGAGCTAGGACAAACA**	457	0.2
	Mug-var-A285	**TAACACGGACCAAACAAAAAGT**		0.2

Primer mix 1 (M1) amplifies DNA of *Mecynargus paetulus, Diplocephalus helleri, Erigone atra, Janetschekia monodon, Entelecara media and Walckenaera vigilax* while primer mix 2 (M2) amplifies DNA of *Erigone tirolensis, Agyneta nigripes, Robertus arundineti* and *Mughiphantes variabilis*. Primer concentrations are the final concentration in the multiplex PCR in M1 and M2. The species column indicates the target species for the primers. Primer names are composed by the species abbreviation and the allocated number, ‘S’ referring to forward primers and ‘A’ to reverse primers.

Samples of identified adults from the species *Erigone tirolensis, E. atra, Mecynargus paetulus, Agyneta nigripes, Mughiphantes variabilis, Walckenaera vigilax, Entelecara media, Janetschekia monodon, Diplocephalus helleri* (Linyphiidae) and *Robertus arundineti* (Theridiidae) were sequenced. Universal invertebrate primers, LOC1490 and HCO2198 [23] were used to amplify approximately 660 base pairs of the 5′ end of the COI gene. Each 10 µl reaction contained; 1x PCR Buffer (GeneCraft, Lüdinghausen, Germany), 0.2 mM dNTPs (GeneCraft), 1 µM of each primer, 5 µg bovine serum albumin (BSA), 3 mM MgCl_2_, 0.375 U Taq polymerase (GeneCraft), 2.5 µl of the spider DNA and RNase-Free water (Qiagen, Hilden, Germany) to adjust the volume. Cycling conditions included an initial denaturation for 2 min at 94°C, 35 cycles with 30 s at 94°C, 30 s at 54°C and 1 min at 72°C followed by one final elongation step of 3 min at 72°C. Negative and positive controls were included on all PCR plates. PCR products were separated and visualised using QIAxcel, an automatic multi-capillary electrophoresis system (Qiagen). Samples were then sent for forward and reverse sequencing of COI PCR products (MWG Eurofins) and manually edited using BioEdit [24].

For some species it was difficult to obtain high quality sequences (strong signals in the electropherogram and clearly distinguishable peaks). Additionally, in some cases such as *E. media* it was not possible to get any sequences with the reverse primer. This meant that not all the sequences used were sequenced in both directions. Another problem was the presence of *Rickettsia* spp. DNA in the samples. This was particularly problematic in *E. tirolensis* where over half of the individuals sampled gave *Rickettsia* spp. sequences. It was therefore necessary to remove theses samples from our study. Attempts were made to target other genes, including 12S rRNA, 18S rRNA, as well as other parts of the COI and COII. Unfortunately, the 18S and 12S sequences were too similar to those of the *Pardosa* spp., while the resulting COII sequences were no better than the COI sequences obtained. To ensure that we were not amplifying nuclear mitochondrial pseudogenes procedures outlined in Song *et al* [25] were followed. When preparing the samples for sequencing we checked for double bands (none were present) and all electropherograms were looked at in detail to ensure that there were no double peaks. If this was the case these samples were re-sequenced or excluded. All sequences were translated into amino acids and checked for stop codons, frame-shifts and high rates of non-synonymous mutations.

Sequences from each individual were used and where possible, sequences from individuals caught in other years in the Rotmoos glacier foreland were also included. The species-specific primers were designed using Primer Premier 5 (Premier Biosoft International, Palo Alto, CA, USA). The most complete of 46 different sequences were used for primer design (see Alignment S1 in Supporting Information). For the species with more than one haplotype, a consensus sequence was created to find an adequate priming site for all the members of this species.

The primers were combined, creating two multiplex PCR systems to screen for all ten species of spider. After testing the primers individually and in different combinations and concentrations in gradient PCR, the system was optimised to amplify the DNA of the different species (see Results section). A non-target test was conducted for both multiplex PCR systems in which 121 non-target specimens [26] were included to test if their DNA was amplified by the Linyphiidae and Theridiidae primers.

DNA-extracts from all field-collected spiders, including juveniles and adults, were tested with the two newly established multiplex PCR assays. Those that gave no positives were retested to see if it was possible to detect any more species-specific DNA. Additionally, all samples that gave no bands after the second screening were tested with the general primers [23] to check for the presence of amplifiable DNA of any kind (extraction success).

### Statistical analysis and data representation

Phylogenetic distance between the species was calculated and a phylogenetic tree created. The objective of the tree was not for phylogenetic reconstruction as this is not the aim of the study, but to visualise the phylogenetic variation for the part of the COI sequence obtained. For this purpose all sequences were aligned using BioEdit [24], then sequences were trimmed to fit the longest sequence possible without losing any of the species. As some of the individuals were hard to sequence, possibly due to the presence of DNA from endosymbionts, the final dataset was composed of 46 sequences each of 367 bp in length (see Alignment S2 in Supporting Information). Genetic distances were calculated using p-distance as it performs equally well as more complex models [27] and for the objective of this study it was adequate. The neighbour-joining tree algorithm [28] was used for the initial tree as this gave the best results when the phylogeny was assessed using biplots and correlation indices. The resulting tree was then optimised using maximum likelihood phylogenetic reconstruction to find the best fitting tree. The tree was rooted using *Pardosa nigra* (Lycosidae), a species also found in the glacier forelands examined here.

To compare the results from the morphological identification and the molecular method, ordinations from both sets of data were used (principal coordinates analyses with Bray-Curtis distances based on square root transformed counts of individuals). These were then compared by Procrustes analysis, which yields a joint ordination pattern. Additionally, a Mantel test (with 9999 randomization runs) for the association between the two distance matrices was performed. The species composition was graphed and Bray-Curtis distances used to investigate the community composition between valleys and succession stages. All phylogenetic analysis and species composition analysis were done in R (R Development Core Team 2012), using packages adegenet [29] and phangorn [30]. CANOCO 5 [31] was used for ordination and Procustes analysis and PC-ORD 6 [32] for the Mantel test.

## Results

### Molecular identification of spiders using multiplex PCR

The two multiplex PCR systems in Table 1 amplified the DNA of the 10 targeted spider species successfully. Each 10 µl PCR reaction contained; 1x QIAGEN Multiplex PCR master mix (Qiagen Multiplex Kit), each primer at its specific concentration (Table 1), 5 µg bovine serum albumin (BSA), 1.5 µl of the sample DNA and RNase-Free water (Qiagen) to adjust the volume. The thermocycling conditions were initial denaturation 15 min at 95°C, 35 cycles with 30 s at 94°C, 3 min at 64°C and 1 min at 72°C and one final elongation stage of 10 min at 72°C. Negative and positive controls were included in all PCRs.

Out of the 121 non-target taxa tested, two samples, one muscid and one bibionid specimen gave PCR products of the size of *E. tirolensis* and *A. nigripes*, respectively. The PCR products for these two samples were sequenced and it was possible to confirm that in both cases the DNA found was DNA of the target species. The most likely explanation to that is that traces of spider DNA were contaminating these non-target samples which were mass-collected in Malaise traps and yellow bowls which linyphiid spiders can potentially enter. Therefore, it was possible to infer that the primers were targeting the spider DNA and not that of the non-target taxa.

While screening, there were instances when more than one amplicon size was generated in the PCR, indicating the consumption of one species by another. In most cases there was a band (i.e., PCR product) that was stronger than the other so it might suggest that the weaker band was the gut content DNA and the stronger band the actual spider. As many of the samples giving two bands were also determined morphologically, it was possible to check if the stronger band coincided with the initial identification. This being the case, the samples were included for further analysis.

**Figure 1:**
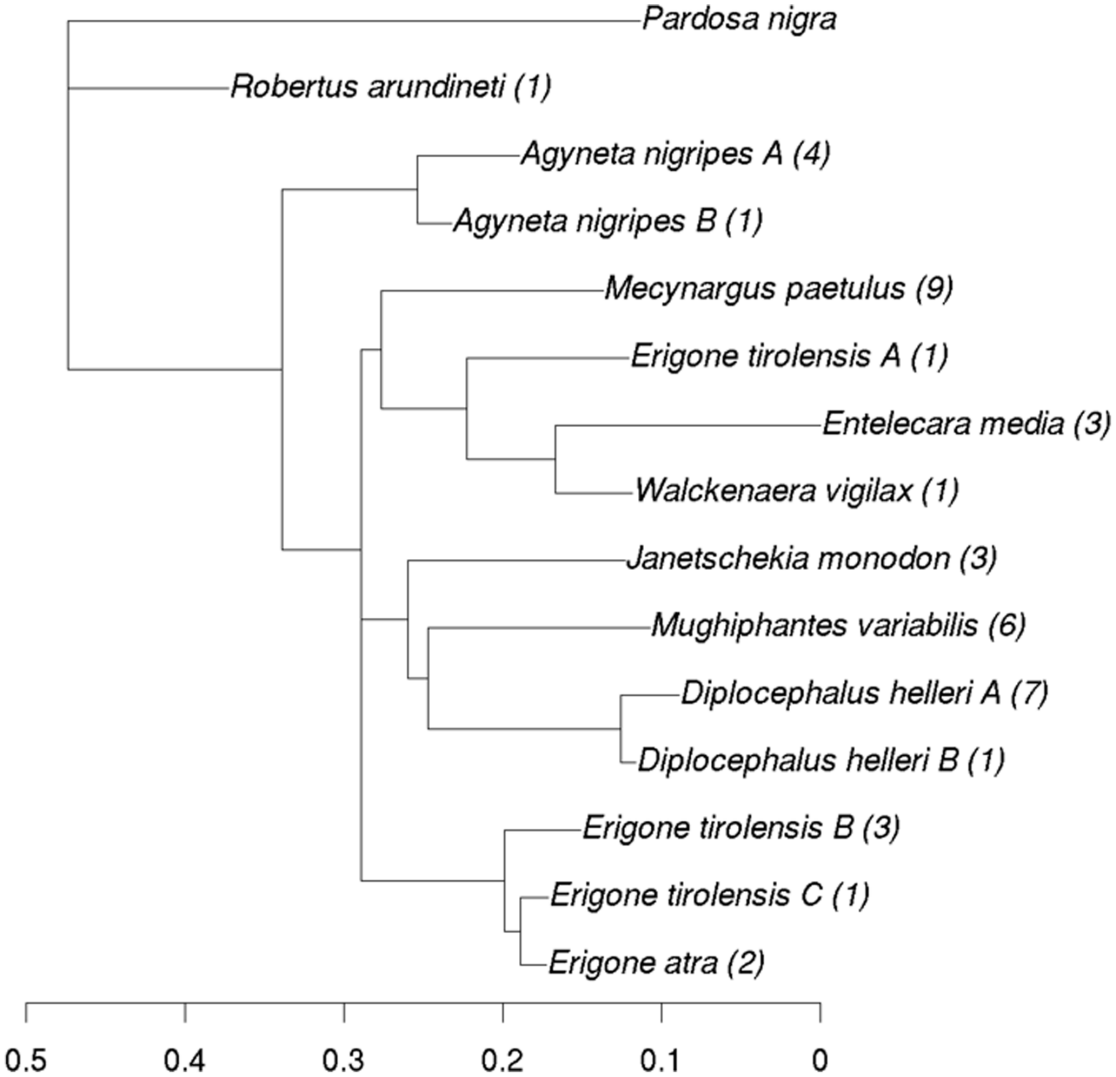
Phylogram showing the main linyphiid species found in the pioneer stages of the three glacier forelands and *Pardosa nigra*. The numbers in brackets are the number of successfully sequenced individuals, providing a specific haplotype. Scale bar indicates substitutions per site.

The phylogram in Fig. 1 shows that clades are consistent with species with the exception of *E. tirolensis*. In this species three different haplotypes occurred: types C and B were similar to *E. atra*, but type A that was represented by a single individual did not share the *Erigone* clade. The COI sequence of the theridiid, *R. arundineti*, was different from all the linyphiid species found in the glacier foreland.

### Comparing morphological and molecular data sets

More than half (55%) of the 735 field-collected linyphiid and theridiid spiders were juveniles and thus not identifiable with traditional morphological methods. After two screenings with the multiplex PCR systems 90% of the linyphiid and theridiid spiders collected in the three glacier forelands were identified. From the remaining 10% of unidentified samples 82% gave positives with the general primers, demonstrating that there was amplifiable DNA in the samples. From the adult spiders that were morphologically identified, 93% were in accordance with the molecular identification.

**Figure 2:**
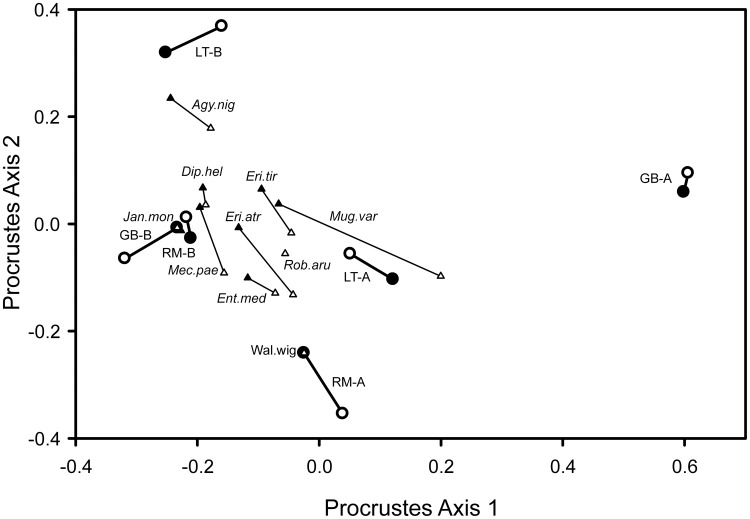
Procrustes analysis of the results of the Correspondence analysis of the morphological and molecular data. Species' centroids are represented by triangles and the areas by circles. Closed shapes are molecular data; open shapes are morphological data. Early pioneer stage (A). late pioneer stage (B). Langtal (LT), Rotmoostal (RM), Gaisbergtal (GB). Species codes: Dip.hel (*Diplocephalus helleri*), Eri.tir (*Erigone tirolensis*), Agy.nig (*Agyneta nigripes*), Mec.pae (*Mecynargus paetulus*), Mug.var (*Mughiphantes variabilis*), Rob.aru (*Robertus arundineti*), Wal.wig (*Walckenaera vigilax*), Jan.mon (*Janetschekia monodon*), Eri.atr (*Erigone atra*), Ent.med (*Entelecara media*).

Ordinations of the linyphiid and theridiid community composition of the three valleys and two succession stages yielded very similar results with the molecular (adults and juveniles) and morphological (adults only) data sets. Procrustes analysis (Fig. 2) gave a disagreement of only 6% between the two ordination patterns of the six sites. The association between the Bray-Curtis distance matrices for the two data sets was highly significant (P = 0.0036, Mantel test). Also the species (centroids calculated manually) appear very similar in the ordinations, the only larger difference was found in *M. variabilis*.

### Linyphiid and theridiid community composition

**Figure 3:**
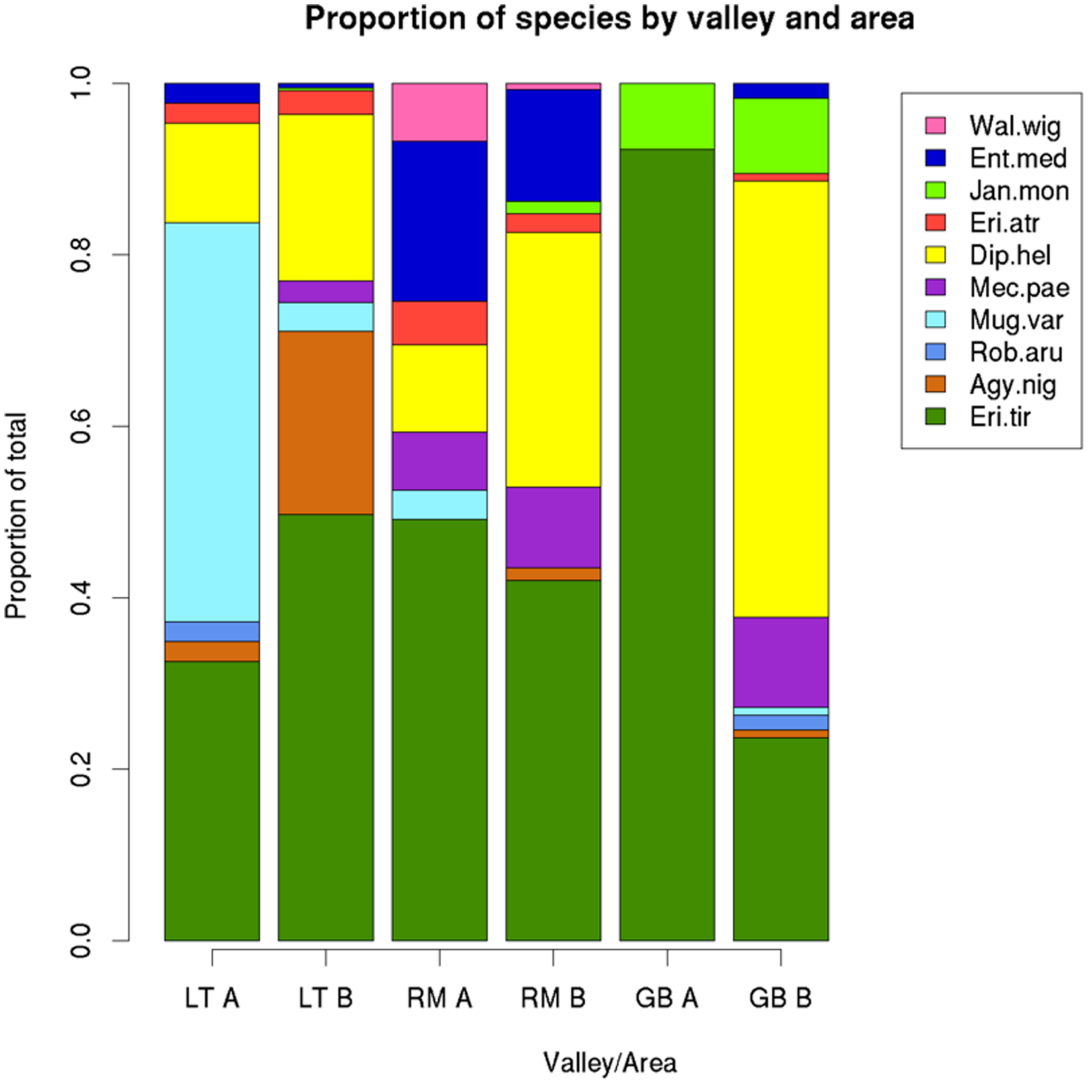
Differences in linyphiid spider species composition between three glacier foreland valleys for spiders collected in early (A) and late (B) pioneer stage. Langtal (LT), Rotmoostal (RM), Gaisbergtal (GB). Species codes: Dip.hel (*Diplocephalus helleri*), Eri.tir (*Erigone tirolensis*), Agy.nig (*Agyneta nigripes*), Mec.pae (*Mecynargus paetulus*), Mug.var (*Mughiphantes variabilis*), Rob.aru (*Robertus arundineti*), Wal.wig (*Walckenaera vigilax*), Jan.mon (*Janetschekia monodon*), Eri.atr (*Erigone atra*), Ent.med (*Entelecara media*).

*Erigone tirolensis* was the species that could be frequently found in both early and late pioneer stages in the three glacier forelands (Fig. 3). *Entelecara media* was also found in all valleys but Gaisbergtal and it was rarely caught in the late successional stage in Langtal. *Diplocephalus helleri* also occurred in relatively high numbers in all areas except in the early pioneer stage in Gaisbergtal. While *A. nigripes* and *M. variabilis* were mostly found in Langtal, *J. monodon* was exclusive to Gaisbergtal and the late pioneer stage in Rotmoostal, while *W. vigilax* was only caught in Rotmoostal. *Robertus arundineti* was the least common species with only a few individuals found in the early pioneer stage of Langtal and the late pioneer stage of Gaisbergtal.

The Bray-Curtis dissimilarity indices for these spider communities varied greatly across glacier forelands and successional stages, ranging from the 0.32 dissimilarity between the early and late pioneer stages of Rotmoostal glacier foreland, to a 0.76 dissimilarity between the early and late pioneer stages in the Gaisbergtal glacier foreland. This can be observed in Fig. 2, in which the later stages are more similar and the early stages are more dissimilar from each other. The greatest similarity was found between the late stages of Gaisbergtal and Rotmoostal (dissimilarity index of 0.22). The most dissimilar sites were the early Gaisbergtal and late Langtal (0.84).

## Discussion

The molecular multiplex PCR identification system proved to be a good way of identifying linyphiid and theridiid spiders. From the 735 spiders tested with the two assays, 87% were identified in the first screening with an extra 3% determined when the negative samples from the first screening were retested. Of the non-identifiable individuals, 82% worked with the general primers, demonstrating that there was amplifiable DNA in the samples. A possible reason for having no results in the multiplex PCR even though amplifiable DNA was present, is that the spider sample belonged to a species not targeted in our assays. In preliminary work in 2009, small numbers of other species were found in the Rotmoos glacier foreland (e.g. one individual of *Erigone dentipalpis*) that were not included in the current multiplex PCR assays. Considering that just over half of the samples tested were juveniles, which could not be identified with morphological methods, this can be considered an efficient and useful application for DNA identification methods.

The 7% difference between the traditional and molecular identification methods could be due to the difficulties encountered with both methods. Errors in morphological identification have been reported in cases where identification was difficult [33], [34]. However, this was likely not the case in our study as all the samples that gave different identifications were clearly distinguishable species. It is interesting to note that Thaler [35] pointed out that for some alpine species there are uncertainties and further clarifications as to the correct identification are necessary. There are also some species where only males have clear morphological characters, which could also lead to problems in identification. Even so, as the comparison between the morphological and molecular results show, this made no difference to the final conclusions on the community composition.

It is not uncommon for spiders to have bacteria associated with them [36]–[38]. Smith *et al*. [39] recognised the effect *Wolbachia* spp. can have on its host's DNA and how it can cause problems for DNA barcoding. In this study *E. tirolensis* and *E. atra* were particularly hard to sequence as most of the sequences obtained were either entirely *Rickettsia* spp. DNA or a mixture of the original species and *Rickettsia* spp. Goodacre *et al*. [40] found meta-populations of *E. atra* infected with *Rickettsia* spp. so there may be a particular association with the species. Bearing in mind that *Wolbachia* spp., *Spiroplasma* spp. and *Cardinium* spp. are also common in spiders [41], [42], it is important to take this into consideration, particularly when working with new species as it can cause much confusion and there is some room for error. Although it is probably not so relevant in our system, fungi such as *Nomuraea* spp. can also be spider pathogens [43]–[45] and recent work suggest that this fungal DNA might be amplified if mitochondrial DNA is targeted [46], [47]. Therefore, it is necessary to double-check and BLAST all sequences to try to ensure their authenticity. Sequencing as many samples as the budget permits will help to guard against complications of this kind.

Genetic variability is also something that has to be considered before deciding to use a DNA-based identification approach. Species such as *E. tirolensis* have a holarctic distribution and they can be found in many different geographical regions (e.g. Alaska, Greenland, Scotland and Austria) [48]. Hence, there is a high probability of genetic variability in the COI barcoding gene. For example, Robinson *et al.* [38] and Muster *et al.* [49] found variability within the mitochondrial COI gene in spider species as was the case in this study. The most variable species in the current study was *E. tirolensis* where three distinct lineages were found. Therefore, further trapping and sequencing efforts would be advisable to shed some light on the high variability of the *E. tirolensis* of the glacier forelands studied.

We found that 15% of our samples gave more than one PCR product when tested. There was a strong possibility for this to occur, as the spiders were not starved before freezing and so the gut content DNA is included in the extraction. Starving the individuals before extraction could help improve the chances of not having these double bands in the multiplex PCR. This may have also been the cause for the two samples from the non-target test containing the target spider DNA, as the specimens used for these samples were not starved or washed before DNA extraction.

Another explanation could be cross contamination of DNA between samples when the spiders were morphologically identified. However, this is highly unlikely due to the precautions taken (cleansing with ethanol and flaming between identifications). In nearly all cases, when two PCR products were obtained from an individual sample, there was a band (i.e. PCR product) that was weaker than the other. As mentioned earlier, we were able to compare some of these results with the morphological identification. These agreed, so it encouraged us to accept the stronger band as the correct identification. Even if we had not included these ‘double’ band results there would have been no difference in the general outcome of the community composition.

The linyphiid and theridiid community composition varied between the three valleys and the two pioneer stage areas investigated in this study. Similar species were found in all the valleys but the proportions were not the same and some species (e.g. *A. nigripes*) seemed to be exclusive to some areas. This could mean that certain species are more specialised and therefore prefer a specific habitat, or it could also mean that they are incoming species from further down the valley, which have not established yet. Similarly, there appeared to be no pattern behind the distribution of species in the pioneer stage areas. When a species was found to be common in an area it was not found concentrated in one point but in a greater or smaller degree over the whole area (data not presented). However, in some cases there were traps that caught more individuals but there appeared to be no particular places within the areas where the same species were found. This could be due to the sampling scale and it might be necessary to sample areas smaller than 1 m^2^ to detect any habitat preference. Gaisbergtal had a distinctively different species composition, with the early pioneer site being particularly species poor and dominated by *E. tirolensis*. It was also the only valley with a high number of *J. monodon* whereas the Langtal and the Rotmoostal were more similar in their linyphiid and theridiid spider community composition.

In conclusion, multiplex PCR-based identification of linyphiid and theridiid spiders can be very advantageous, particularly considering the difficulties in identification of juvenile specimens. We also demonstrated that the data sets derived from the morphological and molecular identification of the linyphiid and theridiid community did not differ significantly. Therefore, identifying juvenile and adult specimens with multiplex PCR assays can be useful for rapid, cost-effective identification of large numbers of specimens. The molecular approach we have presented here will facilitate the identification of Alpine linyphiid and theridiid species, broadening the possibilities of research in these families of spiders.

## Supporting Information

Alignment S1 **Primer alignment.** (Alignment-S1-Primers.fas).
(FAS)

Alignment S2 **All 367 bp sequences.** (Alignment-S2-Seq367.fas).
(FAS)
